# Raccoon Roundworm Eggs near Homes and Risk for Larva Migrans Disease, California Communities

**DOI:** 10.3201/eid0912.030039

**Published:** 2003-12

**Authors:** Gabriel P. Roussere, William J. Murray, Caroline B. Raudenbush, Michael J. Kutilek, Darcy J. Levee, Kevin R. Kazacos

**Affiliations:** * San Jose State University, San Jose, California, USA; † Purdue University, West Lafayette, Indiana, USA

**Keywords:** *Baylisascaris procyonis*, raccoon, roundworm, latrines, zoonoses, larva migrans, baylisascariasis, public health

## Abstract

The raccoon roundworm, *Baylisascaris procyonis*, is increasingly recognized as a cause of serious or fatal larva migrans disease in humans and animals. We assessed the potential for infection in three northern California communities by determining the density and distribution of raccoon latrines, where transmission primarily occurs, and the prevalence of eggs at private residences. We collected fecal samples from 215 latrines and found that 44%-53% of the latrines contained *B. procyonis* eggs and that 16% to 32% contained infective eggs. Among the properties surveyed, 28%-49% harbored at least one latrine that was positive for *B. procyonis* eggs. The latrine densities in these communities were higher than any previously reported. The presence of *B. procyonis* eggs in raccoon latrines was common, widespread, and closely associated with human habitation. Where raccoon densities are high, education of the public and removal of raccoons may be necessary.

The raccoon, *Procyon lotor*, is a free-ranging mammal found throughout urban and rural areas of North America. Raccoons harbor a wide variety of infectious agents and parasites, many of which are zoonotic. One of these, the raccoon roundworm, *Baylisascaris procyonis* (Nematoda: Ascaridoidea) (Figure 1), is a well-known cause of visceral, ocular, and neural larva migrans in humans and other animals (*1*–*3*). Fatal or severe central nervous system (CNS) disease from *B. procyonis* has been reported in >90 species of birds and mammals (*2*); 13 known cases of neural larva migrans were reported in humans, primarily in children <2 years of age (*2*–*11*).

**Figure 1:**
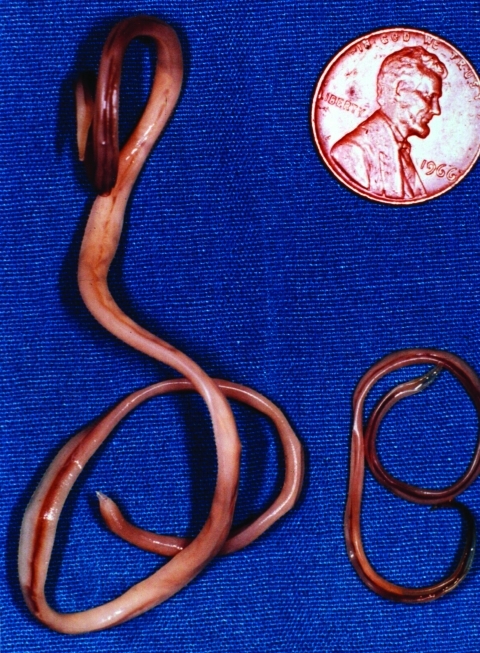
Adult *Baylisascaris procyonis* removed from the small intestine of a raccoon. Adult females (left) are about 24 cm long; males (right) are about 12 cm long. (Reprinted from Clinical Microbiology Newsletter 2002;24:1–7; with permission from Elsevier Science).

The biologic, morphologic, and ecologic characteristics of *B. procyonis* are similar to those of other ascarid parasites of carnivores such as the common canine roundworm, *Toxocara canis* (*1*–*3*). Unless an unusually heavy infection occurs in juvenile raccoons, *B. procyonis* causes little or no clinical disease in its natural host. Like several other ascarids of mammals, *B. procyonis* has a direct or indirect life cycle, depending on the age of the definitive host (*2*). Raccoons become infected in one of two ways: 1) young raccoons become infected by eating eggs during investigative behavior and during feeding and grooming activities with other members of their social group; 2) adult raccoons acquire the infection by ingesting intermediate hosts (rodents, rabbits, birds) infected with the larvae of *B. procyonis* (*2*). In intermediate hosts, CNS disease develops from larval migration, making the hosts easy prey for raccoons. In either case, the life cycle of the parasite is completed after larvae are released in the intestinal tract and develop into adult male and female worms. This process requires approximately 63 days after egg infection and approximately 35 days after raccoons ingest larvae in intermediate host tissues (*2*). Adult female worms in the small intestine of a raccoon collectively may produce millions of eggs per day, which are shed in feces (*1*–*3*). Once outside the body, the eggs become infective (i.e., containa second-stage larva) in approximately 2 to 4 weeks, depending on environmental conditions such as moisture and temperature (*1*–*3*). Like other ascarid eggs, the eggs of *B. procyonis* are resistant to degradation in the environment and can survive for years under appropriate conditions (*1*).

A key feature of the epidemiology of baylisascariasis is the behavior of raccoons. Raccoons habitually defecate in communal sites called latrines. The locations of latrines are associated with various natural and human-made structures (*2*,*12*). In urban and suburban areas, raccoons establish latrines on rooftops, in attics, in and around chimneys, and on other roof protrusions, stumps, woodpiles, decks, and lawns, especially near trees (*1*,*2*) (Figure 2). Where raccoon densities are high, substantial amounts of feces containing large numbers of resistant *B. procyonis* eggs accumulate at latrines, which become long-term focal sources of infection for humans and other animals (*1*,*2*,*13*,*14*). Thus, humans may become infected accidentally by coming into contact with active or abandoned latrine sites and inadvertently ingesting eggs containing infective *B. procyonis* larvae (Figure 3). Young children are especially at risk for infection because of their propensity to handle objects and put them in their mouth.

**Figure 2:**
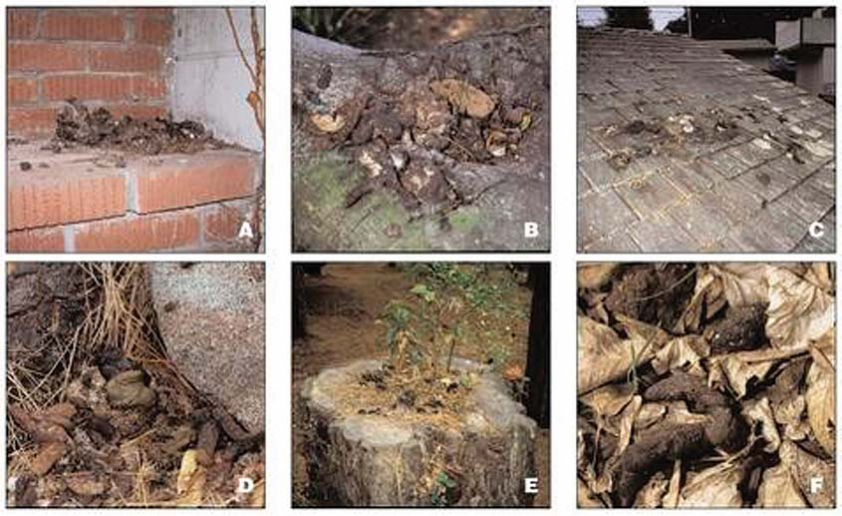
Typical raccoon latrines found in urban/suburban environments. (A) Latrine on a chimney ledge, illustrating the climbing abilities of raccoons and their tenacity in maintaining latrines. (B) Large latrine in the crotch of an oak tree approximately 3.5 m (15 feet) above ground. The sides of the tree were visibly stained with fecal residue that rain had washed down the trunk, contaminating a child’s play area below with *Baylisascaris procyonis* eggs. (C) Large latrine, in use for years on a house roof, unknown to the home owner. (D) Latrine site on the ground near downed timber and rocks in a suburban yard. Note the variety of fecal materials (including seeds, crustacean shells, and human refuse), reflecting the diversity of the raccoon diet. The homogenous-appearing fresh scat in the center is composed of digested pet food. (E) Latrine on a stump in a suburban park with plants sprouting from seeds in the scat. Granivorous birds and mammals are attracted to such locations, as are curious children. (F) Raccoon scat hidden in leaf litter in a suburban back yard, indicating how occult contamination may be.

**Figure 3:**
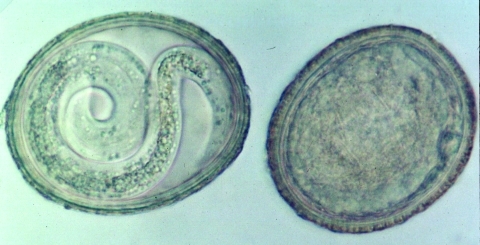
*Baylisascaris* *procyonis* eggs recovered from raccoon feces from a latrine in a playground sandbox. Left, infective egg containing a fully formed larva (40x). Right, an undeveloped or degenerate noninfective egg. *B. procyonis* eggs are ellipsoid, approximately 75 μm x 60 μm in size, with a brown, finely granular surface. (Reprinted from Clinical Microbiology Newsletter 2002;24:1–7; with permission from Elsevier Science.)

Thirteen known cases of *Baylisascaris* encephalitis have occurred in humans in the United States from California, Illinois, Michigan, Minnesota, New York, Oregon, and Pennsylvania. Five of these cases were eventually fatal (*4*,*5*,*9*,*11*). In addition, a 14th case is suspected in a young girl with CNS larva migrans from Missouri (*15*). Since 1993, four documented cases of *Baylisascaris* encephalitis have occurred in California, three involving young boys, and one case in a developmentally delayed 17-year-old boy who had pica. Two of the four cases occurred in northern California, in San Leandro in 1993 (*7*) and Pacific Grove in 1998 (*8*). The other two cases occurred in southern California, in Los Angeles in 2000 (*11*) and in Santa Barbara in 2002 (D. Paul, et al., unpub. data). In addition, a case of *Baylisascaris* ocular larva migrans was identified in a 29-year-old man from Marin County (*16*).

The present study was prompted by the Pacific Grove case, in which an 11-month-old boy became infected with *B. procyonis* and severe neural larva migrans and unilateral ocular disease (*8*) developed. Infective *B. procyonis* eggs were found in raccoon latrines that were numerous on the patient’s property and an adjacent lot. Further investigation indicated large populations of raccoons throughout the area based on sightings, homeowner complaints, and the presence of raccoon latrines in residential areas. All indicators suggested a substantial potential for transmission of *B. procyonis* to humans and animals. However, despite an increasing recognition of baylisascariasis in humans, data are lacking on the distribution of raccoon latrines and the prevalence of *B. procyonis* eggs in areas where humans reside.

We investigated the risk for exposure to *B. procyonis* in three northern California communities by systematically examining raccoon latrines. The purpose of the study was to determine the density and distribution of raccoon latrines and the prevalence of *B. procyonis* eggs located near human habitation.

## Materials and Methods

### Field Methods

We chose three study areas in northern California that were known to have large populations of raccoons. Pacific Grove and Carmel are coastal cities lying close to one another on the Monterey Peninsula. Both communities have had ongoing complaints from residents regarding raccoon depredation to their homes and properties. A recent documented case of baylisascariasis occurred in Pacific Grove (*8*), but none occurred in Carmel. The third community, the Naglee Park neighborhood of San Jose, is approximately 112 km (70 miles) northeast of the Monterey Peninsula. It is an inland community lying in the Santa Clara Valley. Naglee Park has had few complaints from residents regarding raccoon activities and no documented cases of baylisascariasis. The three communities are similar in their demographics, and all have older homes and established plantings of mature trees and shrubs.

We used newspaper advertisements and flyers to bring the study to the attention of the residents. Property owners called a telephone messaging system and agreed to have their properties studied. We mapped the locations of the properties on city maps and found that they approximated a random distribution in all three communities. We assigned unique identification codes to the properties, corresponding to the city and the numerical order in which they were studied. We measured the total area of each property in square meters and systematically surveyed each site for the presence of raccoon latrines.

Latrines were identified by the presence of raccoon feces, which have characteristic size, shape, odor, and other physical attributes; typically, they are dark, attenuated scats, approximately 7- to 15-cm long x 2 cm in diameter, have a pungent odor, and contain a variety of seeds and other food items (*2*,*13*,*17*). Fecal piles <1 m apart were considered to be from the same latrine. We mapped the location of each latrine, measured its diameter, and counted the number of scats it contained. All feces were collected from each latrine or, if the volume of feces was large, representative portions of each recognizable scat were collected and placed in either plastic bags or 50-mL plastic tubes. Typically, the fecal weight from a single latrine ranged from 30 g to 750 g. All samples were examined for *B. procyonis* eggs within 1 to 3 days of collection. Samples that were negative were reexamined.

### Laboratory Methods

We determined the presence of *B. procyonis* eggs in fecal samples by a modified detergent wash flotation procedure using Sheather’s sugar solution, specific gravity 1.25–1.27 (*18*,*19*). In some cases, desiccated samples were rehydrated with distilled water to soften them before processing. Preparations were examined by using a light microscope by bright-field and differential interference contrast methods.

We identified *B. procyonis* eggs (Figure 3) on the basis of their size and other morphologic characteristics (*1*,*2*). Once identified, the eggs were classified as noninfective, if they contained an undeveloped embryo, or potentially infective, if they contained a fully formed larva (*3*).

## Results

**Figure 4:**
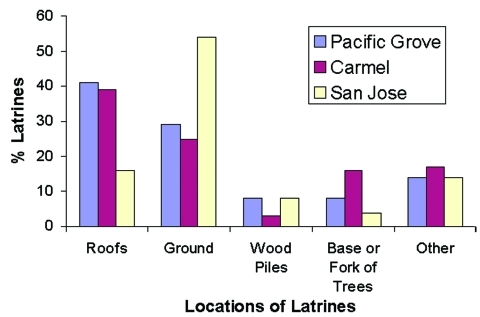
Percentage of raccoon latrines found at various locations in Pacific Grove, Carmel, and San Jose, CA (number of latrines = 244). The “other” category includes window ledges, attics, fences, decks, and so forth.

We found 127 raccoon latrines on 80 properties in Pacific Grove, 64 latrines on 38 properties in Carmel, and 53 latrines on 46 properties in San Jose, for a total of 244 latrines on 164 properties. Property sizes were more variable in Pacific Grove (1,828 + 6,023 m^2^) than in Carmel (777 + 1,263 m^2^) or San Jose (752 + 447 m^2^). The density of latrines was 8.7/hectare (3.5/acre) in Pacific Grove, 21.7/hectare (8.8/acre) in Carmel, and 15.3/hectare (6.2/acre) in San Jose. In Pacific Grove and Carmel, we found most latrines on roofs (39%-41%); in San Jose most were located on the ground (54%) (Figure 4).

**Figure 5:**
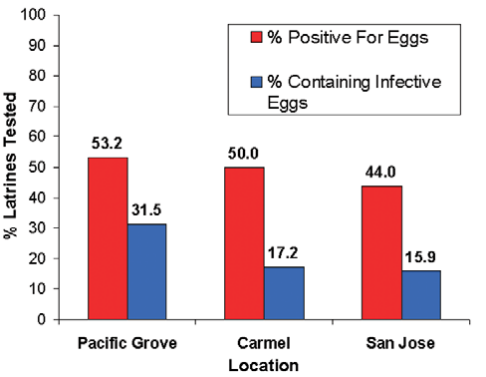
Percentage of raccoon latrines that tested positive for *Baylisascaris procyonis* eggs and those containing potentially infective eggs (number of latrines = 215).

**Figure 6:**
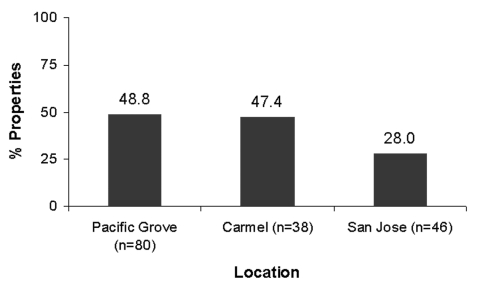
Percentage of properties that contained at least one raccoon latrine positive for *Baylisascaris procyonis* eggs (number of properties = 164).

**Figure 7:**
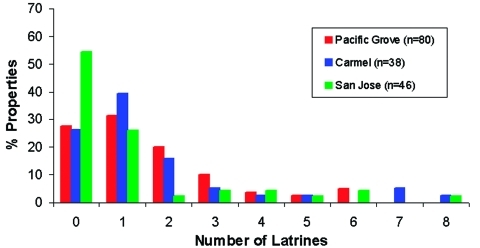
Frequency distributions for the number of raccoon latrines found in Pacific Grove, Carmel, and San Jose, California.

Of the 244 latrines, 29 were not accessible for sampling due to their unsafe locations, e.g., on damaged roofs. We examined the remaining latrines for *B. procyonis* eggs, and approximately half (44.0%-53.2%) of these were positive, with many containing infective eggs (15.9%-31.5%) (Figure 5). Nearly half of the properties in Pacific Grove and Carmel (47.4%-48.8%) and more than one quarter of those in San Jose (28.0%) contained at least one latrine that was positive for *B. procyonis* eggs (Figure 6). Over half of the properties in San Jose (54%) and more than one fourth in Pacific Grove and Carmel (27%-28%) did not harbor latrines. Most of the properties examined had three or fewer latrines, but a few contained as many as eight (Figure 7).

## Discussion

The widespread distribution and high densities of latrines suggest that human and animal contact with raccoon feces and *B. procyonis* eggs is likely to be common in the study areas. However, human clinical infections are uncommon, probably because most persons do not have intimate contact with the sites or exhibit those behaviors (ingestion, pica, geophagia) that would result in heavy infection. Nonetheless, the potential for infection exists, especially for young inquisitive children who might exhibit such behaviors. Moreover, because of the widespread distribution of raccoons, as well as diagnostic difficulties and lack of clinical experience with the spectrum of disease caused by *B. procyonis* in humans, mild or subclinical infections likely go unrecognized. In fact, asymptomatic, low-level infection with *B. procyonis* is probably the most common form of infection (*1*–*3*,*6*).

These study sites had greater densities of latrines and a higher percentage of latrines containing *B. procyonis* eggs than seen in previous studies. Page et al. (*12*) found 42 latrines in an 8.2-hectare woodlot (5.5/hectare) in Indiana and *B. procyonis* eggs in 14% of the latrines sampled. Jacobson et al. (*20*) found 97 raccoon scats in a 280-hectare urban study area and 121 raccoon scats in a 230-hectare rural study area in Indiana (0.35 and 0.53/hectare, respectively). They also found that 27% of the urban scats and 31% of the rural ones contained *B. procyonis* eggs (*20*).

The latrine densities found in our study sites suggest an abundant raccoon population. This conclusion is further supported by field observations of large numbers of raccoons encountered during the surveys. As raccoon density increases, opportunities for intra- and interspecific density-dependent disease transmission also increase. Intraspecific transmission occurs when *B. procyonis* eggs pass from one raccoon to another through social interactions, such as living in the same den and grooming, and by visits to latrines. Interspecific transmission involves intermediate hosts; small granivorous rodents and birds routinely ingest roundworm eggs as they forage for seeds contained in raccoon feces at latrines (*2*,*13*,*14*). The possibility of transmission increases with higher latrine density.

Urban environments typically provide increased food sources and den sites and reduced hunting and predation, leading to higher densities of raccoons (*21*,*22*). Anthropogenic food sources, some inadvertent and others purposefully provided by residents, are important contributing factors to high raccoon densities. Pet food, which is high in protein and fat content, is an important food source for urban and suburban raccoons. In addition, raccoons may forage on fruits and vegetables from gardens and on food wastes from garbage containers (*22*). Numerous potential den sites are found in urban areas, including ground-based decks, crawl spaces under homes, outbuildings, culvert pipes, and chimneys. In the Monterey Peninsula cities (Pacific Grove and Carmel), we observed that mature Monterey pines (*Pinus radiata*) were commonly used as daytime resting sites by urban raccoons.

In rural environments and wooded parks, raccoons tend to prefer raised horizontal surfaces, such as logs, stumps, limbs, and forks of trees as latrine sites (*1*,*2*,*12*). Our results show that accessible roofs of houses and sheds are also common locations for latrines in urban/suburban areas. Many roof latrines in Pacific Grove and Carmel were apparently used for years without the homeowner’s knowledge. These often accumulated substantial amounts of feces. During periods of rainfall, this fecal matter washes down rain gutters to the ground near residences, thus becoming more accessible to family members. Such ground contamination from roof top latrines has been implicated in a recent case of *B. procyonis* neural larva migrans in an 11-month-old child at a day-care facility in Santa Barbara, California (D. Paul, et al., unpub. data).

Ground latrines, which were especially common in San Jose, are also important foci of *B. procyonis* eggs due to their accessibility. Young children (1–4 years of age) frequently have pica or geophagia and often put objects found on the ground in their mouth. They are especially at risk of accidentally ingesting *B. procyonis* eggs and account for most cases of severe infection (*2*,*3*). Moreover, feces on the ground decompose into the surrounding soil, leaving no sign of their presence, but infective eggs released from the feces may remain viable for years (*1*,*2*).

In the late 1990s, both Pacific Grove and Carmel had ongoing raccoon problems, as judged by frequent complaints from citizens. In Pacific Grove, this situation prompted (since 1998) an ongoing program to trap and euthanize nuisance raccoons. Pacific Grove and Carmel have also increased their educational efforts aimed at reducing anthropogenic food sources and shelter for raccoons. Carmel has had no such programs, which may be the reason for the difference in latrine densities between these two cities. The study area in San Jose had few complaints, yet the mean number of latrines per property and the mean density of latrine sites was similar to Pacific Grove and Carmel. The major difference was in the distribution of latrines; San Jose had a greater percentage of properties without latrines, compared with the other two cities, suggesting differences in raccoon density and property usage in this community.

*B. procyonis* can produce devastating neurologic disease in humans, especially young children (*2*–*11*). *B. procyonis* is much more virulent than the dog roundworm, *T. canis*, the most frequently encountered cause of larva migrans in humans (*3*). Clinical signs of CNS infection with *B. procyonis* may develop as soon as 2–4 weeks after ingestion of infective eggs (*23*). Factors influencing the severity of CNS disease in humans and other animals include the number of eggs ingested, the extent and pattern of larval migration in the tissues, especially the CNS, the severity of inflammation caused by migrating larvae, and the amount of tissue necrosis.

Symptoms of baylisascariasis range from varying degrees of mild CNS dysfunction, to severe neural deficits with paralysis, coma, blindness, and death (*2*–*11*,*23*). The larvae of *B. procyonis* have a noted tendency to invade the brain, causing neural larva migrans and ocular larva migrans. Clinical neural or ocular larva migrans is an accidental consequence of somatic migration and larval distribution. Experimental infections in animals have shown that 5%-7% of larvae enter the CNS, but the damage they cause is extensive, attributable primarily to their large size and aggressive migration (*1*–*3*,*23*). Obviously, the greater the number of *B. procyonis* eggs ingested, the more severe the potential clinical problems.

Ingestion of large numbers of *B. procyonis* eggs may produce rapidly fatal neural larva migrans. Despite treatment, progressive neurologic deterioration often continues because of the severe CNS damage and inflammation (*2*,*3*,*23*). Those who survive often have profound neurologic impairment and are severely incapacitated. Their condition may progressively worsen as the brain undergoes postinflammatory atrophy (*3*,*7*,*23*). Diagnosis is often delayed because *B. procyonis* infection is generally not considered during early patient evaluation. This delay contributes to the severity of disease because the larvae continue to migrate unimpeded in CNS tissues, making the prognosis poorer despite later treatment. This infection should be strongly considered in any patient, particularly a young child who has eosinophilic encephalitis, peripheral eosinophilia, and a history of possible exposure. Albendazole and steroids should be administered immediately while serologic and other confirmation of *B. procyonis* infection are sought (*2*,*3*,*7*–*11*).

Human exposure to *B. procyonis* eggs must be prevented, especially in urban and suburban areas where humans and raccoons coexist. Children should be watched carefully when playing in areas with known populations of raccoons and latrines (*3*). If children are seen to ingest raccoon feces from a latrine or other area, albendazole should be administered immediately (25–50 mg/kg/d x 10d; or 400 mg twice a day x 10d), and the raccoon feces sent for examination for *B. procyonis* eggs (*3*). Children should be taught to avoid fecal material, especially from raccoon latrines, and to thoroughly wash their hands after playing outdoors (*3*). Property owners are advised to inspect their properties and homes periodically, including roofs, for latrines.

If latrines are found, determining what is attracting raccoons to the location is important. Raccoons are readily attracted to sources of food, water, and shelter. Misguided persons who actively feed raccoons or leave pet food outdoors may contribute greatly to problems for other property owners in an area. Advice can usually be sought from local animal control or wildlife management agencies to determine what is attracting raccoons to the area and how to take measures to exclude them from taking up residence or establishing latrines in a particular location.

Latrines should be removed promptly and fecal material disposed of properly (*2*,*3*). Wearing rubber gloves, protective overalls, and rubber boots will reduce the possibility of exposure through self-contamination during cleanup activities. If one is working in a confined space such as an attic, a particle facemask should be worn to reduce the possibility of exposure to fungal spores or other contaminants. Feces should be carefully removed, double-bagged in plastic garbage bags, then placed in routine garbage containers for disposal in a landfill or by incineration. If the latrine is located on the ground, approximately 5–7.5 cm of underlying soil should also be removed and discarded.

*B. procyonis* eggs are difficult to destroy without resorting to high heat (e.g., propane gun flame, boiling water, steam) (*2*). (Obviously, using flame sources around a home is hazardous and should be discouraged unless surfaces like concrete or soil are to be decontaminated.) Furthermore, the eggs have a sticky proteinaceous coat that allows them to adhere to surfaces. They can be rendered less sticky by applications of hot water and bleach, which may be useful for removing residual eggs from flammable surfaces. Additional information regarding latrine removal and decontamination can be found elsewhere (*2*), or by contacting appropriate government agencies. Further studies are needed concerning the survivability of *B. procyonis* eggs under varying conditions and the assessment of optimal, situation-specific methods for inactivation.

In areas where raccoon density is high, trapping and removing raccoons may be necessary to decrease depredation and the accumulation of *B. procyonis* eggs in the environment. Municipalities should educate the public about this parasite and the negative effects of providing food and shelter to raccoons and other wildlife. Reducing the availability of anthropogenic food sources is important to decrease populations of raccoons in urban and suburban environments. Coordinated, comprehensive wildlife management practices are recommended. Given the potential for human exposure to this parasite and considering the risk for very young children, the public should be encouraged to adopt practices that will reduce the possibility of infection by *B. procyonis*.
